# BHF177 Suppresses Diabetic Neuropathic Pain by Blocking PKC/CaMKII/ERK1/2/CREB Signaling Pathway through Activating GABA_B_ Receptor

**DOI:** 10.1155/2022/4661519

**Published:** 2022-11-17

**Authors:** Boyu Liu, Fengxi Guan, Jiapeng Zhao, Yao Niu, Hongbo Jiang

**Affiliations:** ^1^Department of Endocrine, The Third Affiliated Hospital of Xinxiang Medical University, Xinxiang 453003, China; ^2^Department of Ultrasonography, Yanggu People's Hospital, Yanggu 252300, China; ^3^Department of Neurosurgery, The Third Affiliated Hospital of Xinxiang Medical University, Xinxiang 453003, China; ^4^Department of Nutrition, The Third Affiliated Hospital of Xinxiang Medical University, Xinxiang 453003, China

## Abstract

The gamma-aminobutyric acid type B (GABA_B_) receptor may participate in the development of diabetic neuropathic pain (DNP). BHF177 serves as a positive allosteric modulator of the GABA_B_ receptor. In the current study, we sought to study the role of the BHF177-GABA_B_ receptor in DNP and its underlying mechanism. Streptozotocin was adopted to induce a rat model of DNP, followed by determination of the paw withdrawal threshold (PWT), paw withdrawal latency (PWL), and glucose level. The effect of BHF177 on DNP by regulating the GABA_B_ receptor in vivo was determined by the injection of BHF177 and/or CGP46381 (a GABA_B_ receptor antagonist) into rat models of DNP. Hippocampal neuronal cells were isolated and cultured, and the neurons and DNP model rats were treated with activators of PKC (PMA), CaMKII (CaCl_2_), or ERK1/2 (EGF) to study the role of GABA_B_ receptors in DNP via regulation of the NR2B-PKC-CaMKII-ERK-CREB pathway. BHF177 suppressed DNP symptoms by activating the GABA_B_ receptors, as evidenced by increased PWT and PWL of DNP rats and the increased number of neurons expressing the GABA_B_ receptor, but this effect was reversed by CGP46381 treatment. BHF177 treatment markedly repressed PKC, CaMKII, p-ERK1/2, and p-CREB expressions in the rat DNP model, but these suppressive effects were abrogated by treatments with PMA, CaCl_2_, or EGF treatment, respectively. To sum up, BHF177 suppresses DNP symptoms by blocking the PKC/CaMKII/ERK1/2/CREB signaling pathway to activate the GABA_B_ receptors.

## 1. Introduction

Diabetes mellitus (DM), one of the most common metabolic disorders, is characterized by poor control of blood glucose levels with prolonged increases, accompanied with reductions in autophagy and endothelial dysfunction [1, 2]. DM can cause multiple serious complications, including diabetic myocardial microvascular injury, diabetic cardiomyopathy, and diabetic neuropathy [3]. Functional damage of the mitochondria has a close relation to the onset of diabetes [4]. Normally, mitochondrial structure and function are regulated by a complex “quality control system,” which encompasses diverse processes including mitochondrial biogenesis, mitophagy, and mitochondria-mediated cell death. Additionally, endothelial mitochondrial fission occurs in some pathological conditions and bears a close relationship to the development and progression of endothelial dysfunction-related conditions, including diabetes-induced complications. The burgeoning increase in the incidence and prevalence of DM around the world brings with it a corresponding increase in the incidence of diabetic neuropathy year by year [3, 5]. Diabetic neuropathic pain (DNP) is the most frequent symptom of diabetic neuropathy; the locus of this pathology is uncertain, but certain evidence implicates the abnormal transmission at the metabotropic GABA_B_ receptors located on glutamatergic neurons of the spinal dorsal horn, which attenuates the inhibition of excitatory glutamic acid released by presynaptic neurons [5, 6]. This will have the net effect of enhancing glutamatergic signaling in dorsal horn neurons, thus activating ascending pathways mediating the sensation of pain. In this scenario, promoting neurotransmission by GABA neurons in the spinal dorsal horn can interfere with pain. However, there have not yet emerged effective treatments for DNP based on this putative mechanism.

The GABA receptor family contains three members, including A, B, and C, all of which are activated by gamma-aminobutyric acid (GABA), one of the chief inhibitory neurotransmitters in the vertebrate central nervous system [7, 8]. The metabotropic GABA_B_ receptors exert repressive actions at multiple regions of the central nervous system and are notably abundant in the superficial dorsal horn of the spinal cord, especially in lamina II [9, 10]. Activation of GABA_B_ receptors in the spinal dorsal horn can inhibit Ca^2+^ channels in presynaptic neurons, then inhibiting their capacity for Ca^2+^-dependent neurotransmitter release [11]. The GABA_B_ receptor on presynaptic neurons not only suppresses thin fiber cells releasing the excitatory neurotransmitter glutamic acid (glutamate) but also exerts feedback regulation of the release of inhibitory neurotransmitters and neuropeptides in the spinal dorsal horn [12]. By this mechanism, the GABA_B_ receptors are thought to participate in antinociceptive effects at the level of the spinal cord.

cAMP response element-binding protein (CREB) is a widely expressed transcriptional factor in the nervous system. Phosphorylation of CREB regulates transcription and translation of diverse genes, with consequences for many signaling pathways [13]. Previous research has shown that phosphorylation of CREB can inhibit the expression of GABA_B_ receptors and promote the expression of subunit NR5B in the NMDA-type glutamate receptor, which may contribute to DNP [14].

In DNP model animals, high glucose levels can induce oxidation stress through advanced glycation end-product (AGE) and NAPDH oxidase, which then causes excessive Ca^2+^signaling and overactivation of protein kinase C (PKC) [15]. PKC/calmodulin-dependent protein kinase II (CaMKII) signaling pathway can then promote phosphorylation of CREB through ERK, thus intensifying DNP. Conversely, activation of the GABA_B_ receptors can significantly repress ERK activity and phosphorylation of CREB, which might alleviate DNP through the PKC/CaMKII/ERK/CREB signaling pathway [13]. BHF177 is positive allosteric modulator of GABA_B_ receptors, with a potential role in treating DNP [16]. In this study, we tested effects and molecular pathways induced by treatment of DNP model rats with BHF177, focusing on the hypothesis that this treatment would alleviate symptoms by inhibition of CREB phosphorylation.

## 2. Material and Methods

### 2.1. Ethical Statement

Animal experiments were conducted with approval of The Third Affiliated Hospital of Xinxiang Medical University. All efforts were made to reduce animal suffering and numbers.

### 2.2. Bioinformatics

The R language “limma” package was used to differentially analyze the diabetic neuropathy-related microarray GSE27382 from the GEO database and screen out the 250 most significantly low-expressed genes. The microarray GSE27382 contains 7 normal samples and 6 diabetic samples. GeneCards was searched to predict diabetic neuropathy-related genes to screen out those with score > 1.5. The intersection of the two results was obtained to identify genes for further study, followed by prediction of downstream pathways.

### 2.3. Construction of a Rat Model of DNP

Streptozotocin (STZ; 50 mg/kg) was adopted to induce a rat model of DNP, as described previously [17]. In brief, Sprague-Dawley (SD) rats (purchased from Laboratory Animal Center of ChongQing Medical University, weight: 250–280 g, age: two months) were injected intraperitoneally (i.p.) with STZ. To improve the success rate of the modeling, STZ was given after 12 h fasting. During the modeling process, rat weight and fasting blood glucose were continuously monitored. Two weeks later, rats with fasting blood glucose > 16.7 mM were confirmed as diabetic rat models, and at one week after STZ injection, pain sensitivity was tested as the paw withdrawal threshold (PWT) using a von Frey needle.

Rats were treated using the up-down method [18]. In brief, rats were placed on a metal mesh and covered with a transparent plexiglass sheet. Following acclimation for 15 min, a series of standardized von Frey hairs was used to vertically stimulate the middle of the rat's hind limbs to provoke retraction into an S shape for 6 to 8 sec. The immediate exhibition of the paw withdrawal latency (PWL) during the stimulation or upon removal of the von Frey Hairs was recorded as a positive response, while a physical withdrawal of the foot was not recorded as a positive reaction until the first positive and negative (or negative and positive) reactions occurred, followed by four consecutive measurements. If the minimum or maximum strength of the von Frey hairs did not provoke a positive response, the 50% PWT of the rat was calculated as 0.25 g or 15 g. The 50% response threshold was interpolated using the formula: 50%threshold = 10 [*X*^f^ + *k*^*δ*^], where *X*^f^ = value (in log units) of the final von Frey hair used; *δ* = mean difference (in log units) between stimuli (here, 0.224); *k* = tabular value for the pattern of positive/negative responses. Rats with less than 4 g 50% PWT were regarded as a meeting criterion for the DNP model.

Thermal hyperalgesia of the rats was assessed using a Hargreaves Apparatus (Ugo Basile, Varese, Italy) according to methods in a previous study [19]. Each measurement was repeated 3 times with the mean value calculated.

### 2.4. Grouping and Treatment of Rats

There were 80 DNP-modeled rats and 10 control rats. Two weeks after STZ injection, rats received pentobarbital sodium (40 mg/kg, i.p.) and the skin, supraspinous, and interspinous ligaments were separated layer by layer under sterile conditions to expose the L4-5 space. Then, PE-10 tubes (length 3–5 cm) were placed into the spinal subarachnoid space. The outer hole was sealed and fixed under the skin after the typical tail swings occurred or cerebrospinal fluid drained out. After the rats recovered from anesthesia, they were observed for two days; rats without lower limb paralysis or other motor impairment were then injected with 20 *μ*L of 2% lidocaine along the outer hole with a 25 *μ*L microsyringe to observe the effect on the lower limb motor function. A paralyzed state indicated the successful insertion of the tubes in the sheath. After another 5 d of recovery, we performed drug administration. During the modeling process, those rats without hyperglycemia, those failing the behavioral analgesia test, and those with unconfirmed implantation of the cannula in the dorsal horn of the spinal cord were excluded, which left a total of 60 successfully modeled rats (75% success rate). The schematic diagram of the experimental process is shown in Figure S1.

The successfully modeled rats were injected with BHF177 (1.5 mL/kg/day, at a concentration of 0.1 g/mL) and/or CGP46381 (0.2% (100 *μ*L containing 200 *μ*g), Sigma, St. Louis, MO, USA), the PKC-specific activator phorbol-12-myristate-13-acetate (PMA) (20 ng/mL), CaMKII-specific activator (CaCl_2_) (100 mM), or ERK1/2-specific activator epidermal growth factor (EGF) (25 ng/mL). Then, we performed 7 d of intramyelinic administration, with the two drugs given with a 15 min interval. The PWT and PWL were tested 30 min after the administration at the seventh day.

A week after drug administration, molecular biology tests were performed. Rats with PWT < 4 g were selected for these experiments. mRNA at the spinal dorsal horn lamina II plate was directly and quickly extracted, and reverse transcription quantitative polymerase chain reaction (RT-qPCR) was used to determine the mRNA expression of GABA_B_ receptors, as well as the PKC and CaMKII. The protein at the spinal dorsal horn laminus II plate was extracted for determining the expression of GABA_B_ receptors using western blot analysis.

### 2.5. Immunohistochemistry

The spinal dorsal horn II lamellar tissue was removed, fixed in formalin for 1 day, dehydrated with 30% sucrose for 2 days, embedded in O.C.T., and cut into sections (15 *μ*m). Then, the sections were blocked with normal sheep serum for 1 h at room temperature and incubated using the primary antibodies for 1 h, along with fluorescent-labeled secondary antibody for 1 h. After mounting, fluorescent cells in the spinal dorsal horn were photographed under a fluorescence microscope, and the number of fluorescence cells was counted in three nonoverlapping fields of view using Image-Pro Plus 6.0 software. The used antibodies (Invitrogen, USA) were GABA_B_ (#PA5-27725, 1 : 1000), NR2B (#71-8600, 1 : 1000), CaMKII (#MA1-048, 1 : 100), PKC (#700043, 1 : 500), p-ERK (#44-680G, 1 : 100), and p-CREB (#MA5-11192, 1 : 400).

### 2.6. Culture and Treatment of Hippocampal Neuronal Cells

Healthy newborn SD rats aged 5–7 d were decapitated following skin sterilization with 75% ethanol, and placed in a precooled D-Hank's medium supplemented with penicillin-streptomycin. The scalp was incised, and the skull was removed. The cerebellar tissue was excised and placed in another prechilled D-Hank's-filled container. Superficial blood vessels and membranes were carefully removed, and the brain tissue was transferred to a centrifuge tube. The tissue samples were added with 0.25% trypsin and placed at 37°C, 5% CO_2_ incubator for 10 min. The cell suspension was aspirated, and the digestion was terminated. The remaining tissue block was trypsinized for another 10 min, and the digestion was terminated by addition of serum-containing culture medium. Cells were gently triturated more than ten times and allowed to stand for 20 s. Then, the above supernatant and cell suspension were centrifuged at 1000 r/min at 4°C for 5 min. After discarding the supernatant, the cells were suspended in culture medium. After cells had adhered to the wall for 30 min in a 37°C incubator, the cell suspension was carefully aspirated. Living cells in the medium were counted using Trypan blue staining and inoculated in a PLL-precoated culture plate with the cell density adjusted to 5 × 10^5^ cells/well. The medium was renewed with maintenance medium on the next day. Then, the Ara-C (final concentration of 10 *μ*M) was added on day 3 to inhibit nonneuronal hyperproliferation. Twenty-four hours later, the solution was renewed. Matured neurons at days 6–8 in culture were used for subsequent experiments.

The GABA_B_ receptor agonist baclofen (10 *μ*M), GABA_B_ receptor antagonist CGP46381 (2 *μ*g/*μ*L), PMA (5 *μ*L), CaCl_2_ (20 *μ*M), and EGF (20 *μ*L) solutions were frozen at −80°C. Neuronal cells were treated with BHF177, BHF177+baclofen, BHF177+CGP46381, BHF177+PMA, BHF177+CaCl_2_, or BHF177+EGF, and cells were subjected to immunoblotting and RT-qPCR.

### 2.7. RT-qPCR

The total RNA was extracted by PureLink^®^ RNA Mini Kit (15596026, Invitrogen, CA, USA), with its concentration assayed utilizing a spectrophotometer, and its purity determined according to the ratio of the photometer at 260 nm/280 nm. cDNA was reversely transcribed by the Superscript^®^ VILOTM cDNA Synthesis Kit (Life Technologies, NSW, Australia). The TaqMan^®^ Gene Expression Assays (Applied Biosystems, Foster City, USA) was adopted to prepare a RT-PCR reaction system in a total volume of 20 *μ*L. RT-qPCR was performed by LightCycler^®^ 480 (Roche, Penzberg, Germany). Glyceraldehyde-3-phosphate dehydrogenase (GAPDH) or U6 were used as internal references, and relative expression of genes was analyzed by 2^−*ΔΔ*Ct^ method. The primer design is shown in Supplementary Table 1.

### 2.8. Western Blot Analysis

The tissue samples were lysed with RIPA lysate containing protease inhibitor and phosphatase inhibitor (Beyotime). The protein concentration was determined using a DC protein analysis kit (500-0111; Bio-Rad, Hercules, CA, USA) at 750 nm. The proteins were separated by sodium dodecyl sulfate polyacrylamide gels and transferred to polyvinylidene fluoride membrane. The membrane was then blocked with 5% bovine serum albumin for 1 h and probed with rabbit anti-human antibodies (1 : 1000) overnight at 4°C. Next, the membrane was reprobed with secondary antibody (1 : 5000). Luminata Western horseradish peroxidase (HRP) substrate (Millipore, Billerica, MA, USA) and Kodak XBT-1 negatives were adopted for color development. The band was quantified using Bio-Rad Quantity One software, normalized to actin level. All results were normalized with blank control values of 100%.

The antibodies used were as follows: GABA_B_ receptor (Invitrogen, #PA1-32248), NR2B (Abcam, Cambridge, UK; ab81271), PKC (Abcam, ab31), CaMKII (Abcam, ab171095), phosphorylation (p)-mitogen-activated protein kinase (ERK1/2) (Abcam, ab54230), p-CREB (Abcam, ab32096), and beta-actin antibody (Abcam, ab8226). Secondary antibody was mouse anti-rabbit immunoglobulin G (IgG) light chain (Abcam, ab8226).

### 2.9. Statistical Analysis

Statistical analyses were conducted by SPSS 21.0 (IBM Corp., Armonk, New York, USA). Measurement data were expressed as mean ± standard deviation of three independent tests. Data comparison among multiple groups was analyzed by one-way analysis of variance, followed by using Dunnet's *t* test. *p* < 0.05 indicated that the difference was statistically significant.

## 3. Results

### 3.1. BHF177 Attenuates DNP by Regulating GABA_B_ Receptor

**Figure 1:**
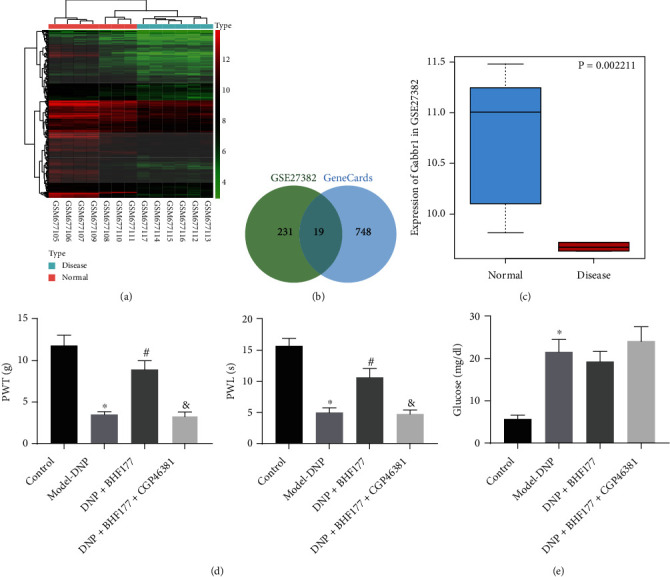
BHF177 confers protection against DNP by regulating GABA_B_ receptor. (a) The expression heat map of the 250 genes with the most significantly low expression in the microarray GSE27382. (b) The intersection of 250 lowest-expressed genes from the microarray GSE27382 and diabetic neuralgia-related genes predicted by GeneCards, showing 19 intersection genes. (c) The expression box plot of GABBR1 in the microarray GSE27382; the blue box indicates the expression of normal samples, and the red box indicates the expression of diabetic samples. (d) The PWT and PWL of DNP rats treated with BHF177 and/or CGP46381. (e) The plasma glucose level of DNP rats treated with BHF177 and/or CGP46381. *n* = 10. ^∗^*p* < 0.05 vs. normal rats, ^#^*p* < 0.05 vs. rat models of DNP, and ^&^*p* < 0.05 vs. DNP rats treated with BHF177. Animal, *n* = 10.

Through the differential analysis of GSE27382, the 250 genes with the significantly lowest expression were obtained (Figure 1(a)). Besides, 767 disease-related genes were retrieved from GeneCards using the key word “Diabetic neuralgia,” and 19 important genes were obtained after the intersection (Figure 1(b)), among which one was the GABA_B_R1 subtype of the GABA_B_ receptor. The GABA_B_ receptor was previously implicated in the hyperactivity of spinal dorsal horn neurons and DNP [20]. According to the data from GSE27382, GABBR1(GABABR1) had a low expression in DNP (Figure 1(c)). To determine the role of BHF177 and GABA_B_ receptors in DNP, we constructed the rat models of DNP and determined their PWT and PWL (Figure 1(d)). Compared with normal rats, rats with DNP exhibited much lower PWT and PWL. Meanwhile, increased PWT and PWL were observed in DNP rats treated with BHF177, but this trend was reversed by CGP46381 treatment, indicating that BHF177 attenuated DNP by regulating the GABA_B_ receptor. Moreover, plasma glucose levels (Figure 1(e)) were also determined, showing that DNP-modeled rats exhibited hyperglycemia. However, BHF177 or BHF177+CGP46381 treatment exerted no significant effect on the glucose level of DNP model rats. Thus, BHF177 relieved DNP in rats through GABA_B_ receptors but had no direct effect on blood glucose levels.

### 3.2. BHF177 Increases the Number of Cells Positive for the GABA_B_ Receptor in DNP Model Rats

**Figure 2:**
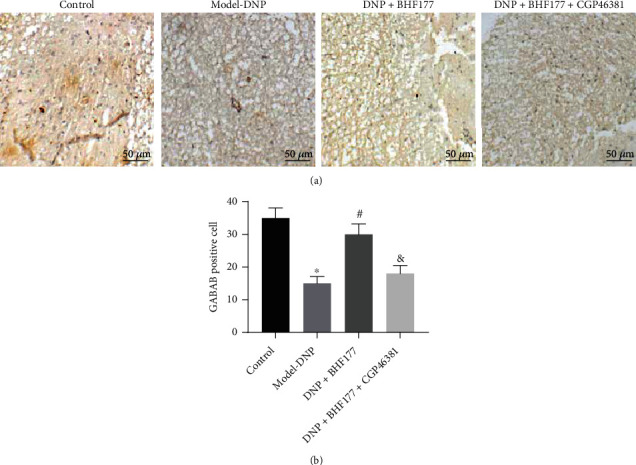
BHF177 increases the number of neurons positive for the GABA_B_ receptor in DNP rats. (a) The number of cells positive for the GABA_B_ receptor in DNP rats determined using immunohistochemistry (×200). (b) Statistic analysis of (a). ^∗^*p* < 0.05 vs. normal rats, ^#^*p* < 0.05 vs. DNP rats, and ^&^*p* < 0.05 vs. DNP rats treated with BHF177. *n* = 10 animals per group.

Immunohistochemistry was adopted to determine the number of neurons positive for the GABA_B_ receptor in the dorsal horn of the rat spinal cord (Figure 2). Compared with the normal rats, there were markedly fewer cells positive for the GABA_B_ receptor in DNP rats. The number of GABA_B_ receptor-positive neurons in DNP rats increased upon BHF177 treatment, while this effect was reversed by CGP46381 treatment (Supplementary Table 2).

### 3.3. BHF177 Regulates GABA_B_ Receptor by Blocking the NR2B-PKC-CaMKII-ERK-CREB Pathway

**Figure 3:**
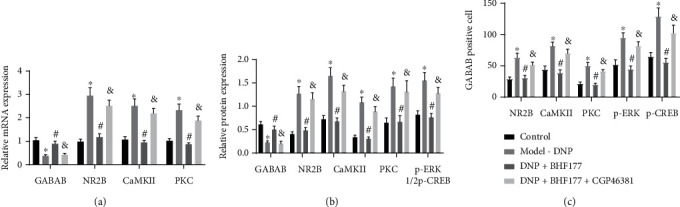
BHF177 targets the GABA_B_ receptor and NR2B NMDA subunit by inhibiting the PKC-CaMKII-ERK-CREB pathway in DNP rats. (a) The mRNA expression of GABA_B_ receptors, NR2B, PKC, or CaMKII in spinal dorsal horn tissues of DNP rats determined using RT-qPCR. (b) The protein expression of GABA_B_ receptors, NR2B, PKC, CaMKII, p-ERK1/2, or p-CREB in spinal dorsal horn tissues of DNP rats determined using immunoblotting. (c) Immunohistochemistry of the number of cells positive for related markers in rat spinal dorsal horn tissue. *n* = 10. ^∗^*p* < 0.05 vs. normal rats, ^#^*p* < 0.05 vs. rat models of DNP, and ^&^*p* < 0.05 vs. DNP model rats treated with BHF177. *n* = 10 rats per group.

The PKC signaling pathway has been proposed to be involved in neuronal damage associated with DNP [21], and CaMKII is also implicated in the abnormal neurotransmission seen in diabetic neuropathy [22]. To understand better the downstream regulatory mechanism, we adopted RT-qPCR and immunoblotting to determine the mRNA and protein expressions of GABA_B_, NR2B, PKC, or CaMKII in DNP rats. Results from RT-qPCR demonstrated markedly downregulated GABA_B_ receptors but upregulated NR2B, PKC, and CaMKII mRNA expressions in DNP rats compared with normal rats. BHF177 treatment markedly elevated GABA_B_ receptors but repressed NR2B, PKC, and CaMKII expression in DNP rats, but this effect was reversed upon CGP46381 treatment (Figure 3(a)). Besides, immunoblotting revealed that DNP rats had decreased GABA_B_ receptors but markedly upregulated NR2B, PKC, and CaMKII expressions as well as increased p-ERK1/2 and p-CREB expressions compared with normal rats. BHF177 treatment activated GABA_B_ receptor expression but markedly repressed NR2B, PKC, and CaMKII expressions as well as that of p-ERK1/2 and p-CREB in DNP rats, but opposite effects were seen upon CGP46381 treatment (Figure 3(b)). Moreover, results of immunohistochemistry matched the immunoblotting results (Figure 3(c)). Thus, BHF177 regulated GABA_B_ receptor expression by negatively regulating the NR2B-PKC-CaMKII pathway in rats with DNP.

### 3.4. BHF177 Blocks NR2B-PKC-CaMKII-ERK-CREB Pathway In Vitro

Based on the above findings, we formed a speculation that BHF177 activated GABA_B_ receptors to block the PKC-CaMKII pathway, thus attenuating DNP symptoms. To test this hypothesis, we treated neuronal cells with BHF177, baclofen, CGP46381, PMA, CaCl_2_, or EGF, first focusing on baclofen and CGP46381. As indicated by RT-qPCR, BHF177 treatment elevated GABA_B_ mRNA expression but inhibited NR2B, PKC, and CaMKII mRNA expressions when compared with control neuronal cells; we saw the same effects when comparing the BHF177 group with the BHF177+baclofen group. Besides, relative to the BHF177 treatment, BHF177+CGP46381 treatment led to decreased GABA_B_ receptor mRNA expression but increased NR2B, PKC, and CaMKII mRNA expressions (Figure S2A). Immunoblotting also revealed that BHF177 treatment elevated GABA_B_ receptor protein expression but inhibited NR2B, PKC, CaMKII, p-ERK1/2, and p-CREB expressions when compared with control neuronal cells; we saw the same effects when comparing the BHF177 group with the BHF177+baclofen group. Besides, relative to the BHF177 treatment, BHF177+CGP46381 treatment led to decreased GABA_B_ receptor protein expression but increased NR2B, PKC, CaMKII, p-ERK1/2, and p-CREB expressions (Figure S2B).

**Figure 4:**
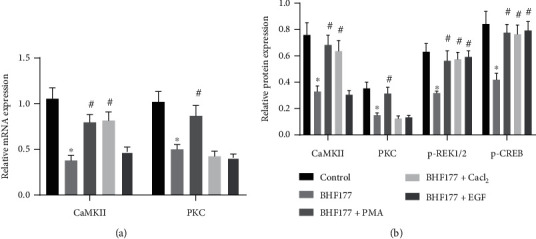
BHF177 inactivates the NR2B-PKC-CaMKII-ERK-CREB pathway in vitro. The neurons were treated with BHF177, PMA, CaCl_2_, or EGF. (a) The mRNA expression of PKC and CaMKII in neurons determined using RT-qPCR. (b) The protein expression of PKC, CaMKII, p-ERK1/2, or p-CREB in neurons determined using immunoblotting. ^∗^*p* < 0.05 vs. normal neuronal cells, ^#^*p* < 0.05 vs. neurons treated with BHF177.

When the downstream genes were activated with different agonists, we found that, compared with the BHF177 group, the BHF177+PMA group showed elevated mRNA expression of PKC and CaMKII in neuronal cells, while the BHF177+CaCl_2_ group had upregulated CaMKII expression but no change in PKC expression. In the BHF177+EGF group, there was no difference in the mRNA expression of PKC and CaMKII (Figure 4(a)). We performed immunoblotting to determine the protein expression of PKC, CaMKII, p-ERK1/2, and p-CREB in neurons (Figure 4(b)). Relative to the BHF177 group, there was increased protein expression of PKC, CaMKII, p-ERK1/2, and p-CREB in neurons of the BHF177+PMA group, while the BHF177+CaCl_2_ group had upregulated CaMKII, p-ERK1/2, and p-CREB expressions but no change in PKC expression. In the BHF177+EGF group, there was elevated expression of p-ERK1/2 and p-CREB, but no difference in the protein expression of PKC and CaMKII. Taken together, these results showed that BHF177 blocked the PKC-CaMKII pathway to inhibit the phosphorylation of ERK-CREB protein.

### 3.5. BHF177 Activates GABA_B_ Receptor to Attenuate DNP by Blocking PKC-CaMKII-ERK-CREB Pathway

**Figure 5:**
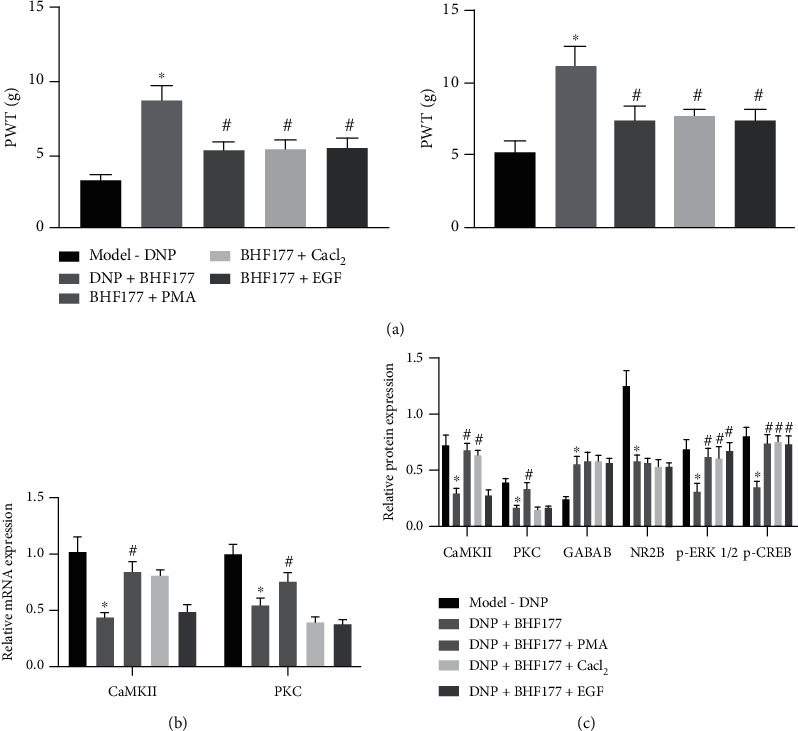
BHF177 activates the GABA_B_ receptor to attenuate DNP symptoms by inactivating the NR2B-PKC-CaMKII-ERK-CREB pathway. DNP rats were injected with BHF177+PMA, BHF177+CaCl_2_, and BHF177+EGF (*n* = 6/group). (a) The PWT and PWL of DNP rats after different treatments. (b) The mRNA expression of PKC or CaMKII in DNP rats determined using RT-qPCR. (c) The protein expression of GABA_B_ receptors, NR2B, PKC, CaMKII, p-ERK1/2, or p-CREB in DNP rats determined using immunoblotting. *n* = 6. ^∗^*p* < 0.05 vs. DNP rats ^#^*p* < 0.05 vs. DNP rats treated with BHF177.

To verify further that the PKC-CaMKII-ERK-CREB pathway was involved in the mechanism of BHF177 in alleviating DNP in rats, the model rats were injected with BHF177+PMA, BHF177+CaCl_2_, and BHF177+EGF. As reflected by Figure 5(a), compared with the DNP rats, DNP rats with BHF177 treatment had increased PWT and PWL behaviors. Relative to the DNP+BHF177 group, the BHF177+PMA, BHF177+CaCl_2_, and BHF177+EGF groups showed decreased PWT and PWL. In the subsequent experiments, RT-qPCR was used to determine the mRNA expression of PKC and CaMKII; results revealed that, compared with the DNP rats, DNP rats with BHF177 treatment had decreased PKC and CaMKII mRNA expression. Relative to the DNP+BHF177 group, the BHF177+PMA group showed elevated PKC and CaMKII mRNA expressions; the BHF177+CaCl_2_ group showed elevated CaMKII mRNA expression but no change in PKC expression, whereas the BHF177+EGF group showed no difference in the mRNA expression of PKC and CaMKII (Figure 5(b)).

Immunoblotting demonstrated that, compared with the untreated DNP rats, DNP rats with BHF177 treatment had elevated protein expression of GABA_B_ receptors but reduced protein expression of PKC, CaMKII, NR2B, p-ERK1/2, and p-CREB. Relative to the DNP+BHF177 group, we found increased protein expression of PKC, CaMKII, p-ERK1/2, and p-CREB but no change in GABAB and NR2B expressions in the BHF177+PMA group. The BHF177+CaCl_2_ group had upregulated CaMKII, p-ERK1/2, and p-CREB expressions but no change in GABA_B_ receptor, NR2B, and PKC expressions, whereas the BHF177+EGF group showed elevated expression of p-ERK1/2 and p-CREB but no difference in the protein expression of GABA_B_ receptors NR2B, PKC, and CaMKII (Figure 5(c)). The aforementioned results demonstrated that BHF177 activated GABA_B_ receptors to attenuate DNP by blocking the PKC-CaMKII-ERK-CREB pathway.

## 4. Discussion

**Figure 6:**
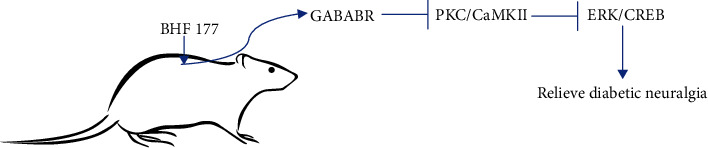
Schematic map of the regulatory role of BHF177 in DNP. BHF177 suppresses NMDA receptor NR2B subunit expression by activating GABA_B_ receptors to block the PKC/CaMKII/ERK1/2/CREB signaling pathway, thus inhibiting DNP.

Over 50% of diabetic neuropathy patients suffer from DNP, a syndrome that includes painful burning or electric shock sensations in the lower limbs, feelings of swelling of the feet or coldness of the legs, and even allodynia [23], much to the detriment of DNP sufferers' quality of life. Although there is a consensus that hyperglycemia is the critical cause of DNP onset, many other factors may play various roles [24]. High blood glucose is always accompanied by multiple metabolic disorders like decreased insulin secretion, imbalance of the cellular ratios of NAD^+^ or NAPD^+^, and indicative of oxidative stress. Therefore, besides hyperglycemia, polyol pathway hyperactivity, oxidative and nitrosative stress, ion channel changes, central pain sensitization, and brain plasticity are among the various pathophysiological mechanisms of DNP development that merit further investigations [5]. Medical treatment can bring a 50% of reduction in the pain level experienced by DNP [25]; main lines of approved treatments include antidepressants, anticonvulsants, and opioids [26, 27]. In the present research, we uncovered the mechanism whereby the allosteric modulator of GABA_B_ receptors BHF177 alleviated DNP. We found that BHF177 activated GABA_B_ receptors, ultimately to suppress phosphorylation of CREB through the PKC/CaMKII/ERK1/2 signaling pathway (Figure 6).

First, we found that allosteric modulator BHF177 could upregulate the expression of GABA_B_ receptors in neuronal culture and that BHF177 treatment could significantly alleviate DNP. GABA_B_ receptor agonists like baclofen were first developed as anxiolytic agents. However, baclofen yielded inconsistent findings of efficacy and intrusive side-effects such as sedation, hypothermia, and excessive muscle relaxation [28]. Surprisingly, positive allosteric modulators (PAMs) of the GABA_B_ receptor show fewer side effects than baclofen, which is encouraging for their possible use to treat anxiety [29]. Unlike baclofen, which binds to GABA_B_ receptor subunit 1, PAMs such as CGP7930 and BHF177 interact with the transmembrane region at the interface of GABA_B_ receptor subunits 1 and 2 [28]. As in anxiety models, BHF177 can significantly reduce the reinforcing and motivation properties of ethanol by targeting the GABA_B_ receptor in alcohol-preferring rats, which may be mediated by suppression of excitatory transmission [30]. In C57BL/6J mice, BHF177 treatment likewise reduced the rate of ethanol self-administration in the form of beer [31]. There has hitherto been no study reporting the effects of BHF on GABA_B_ receptor expression. Our data showed that the abundance of GABA_B_ receptors in the dorsal horn of spinal cord doubled after BHF177 treatment compared with control model rats. Considering the interaction of BHF177 and transmembrane region of GABA_B_ receptors, we speculated that BHF177 might enhance stability of the receptor residing in the membrane, thus extending its lifespan. Confirming this proposition shall call for further experiments. Collectively, we find that BHF177 specifically alleviates DNP through upregulating GABA_B_ receptors.

Second, we find that upregulation of GABA_B_ receptor by BHF177 can inhibit the PKC/CaMKII/ERK/CREB signaling pathway, which finally relieves DNP. The GABA_B_ receptor is a member of G-protein coupled receptor family, which can regulate downstream factors through phosphorylation [32]. PKC has been inferred to be involved with diabetic complications like peripheral neuropathy, notably with respect to the PKC-beta isoform ruboxistaurin (RBX) [33]. Furthermore, TRPV1 activation is blocked by suppressing the PKC signaling pathway, which ultimately causes neuronal damage and DNP [21]. CaMKII is also implicated in the neurotransmission defects in diabetic neuropathy, which is indicated by findings that CaMKII inhibitors successfully suppressed the expression of CaMKII alpha and attenuated nociceptive behavior in model rats [22]. The ERK pathway and related downstream regulators like CREB have been linked to animal models of depression; inhibition of the ERK pathway evokes depression-like behavior in rats, which can be alleviated by treatment with a variety of antidepressants targeting ERK activity [34]. Furthermore, the activities of PKC, CaMKII, ERK, and CREB were all downregulated after BHF177 treatment, indicating that BHF177 functions through this classical signaling pathway to regulate the development of the DNP model.

To sum up, we found in the study that treatment with BHF177 could relieve DNP symptoms in rat models. Furthermore, BHF177 could downregulate the PKC/CaMKII/ERK/CREB signaling pathway via a GABA_B_ receptor coupled signal transduction pathway. Considering its mild side-effect profile and signs of efficacy in the present rat model, we propose that BHF177 shows promise as a clinical medication for DNP.

## Data Availability

The data and materials of the study can be obtained from the corresponding author upon request.

## Supplementary Materials

Supplementary Materials Figure S1: the schematic diagram of the experimental process. Figure S2: BHF177 affects the NR2B-PKC-CaMKII-ERK-CREB pathway in rat cerebellar nerve cells through GABA_B_. Supplementary Table 1: qRT-PCR primer sequence. Supplementary Table 2: the number of positive cells in GABA_B_ receptor.
