# Supplementary material for: BHF177 Suppresses Diabetic Neuropathic Pain by Blocking PKC/CaMKII/ERK1/2/CREB Signaling Pathway through Activating GABAB Receptor
Source: Oxid Med Cell Longev. 2022 Nov 17;2022:4661519. doi: 10.1155/2022/4661519 (PMC9691330; doi:10.1155/2022/4661519)
Supplement: Supplementary Materials — Figure S1: the schematic diagram of the experimental process. Figure S2: BHF177 affects the NR2B-PKC-CaMKII-ERK-CREB pathway in rat cerebellar nerve cells through GABAB. Supplementary Table 1: qRT-PCR primer sequence. Supplementary Table 2: the number of positive cells in GABAB receptor. [file 4661519.f1.docx]

**
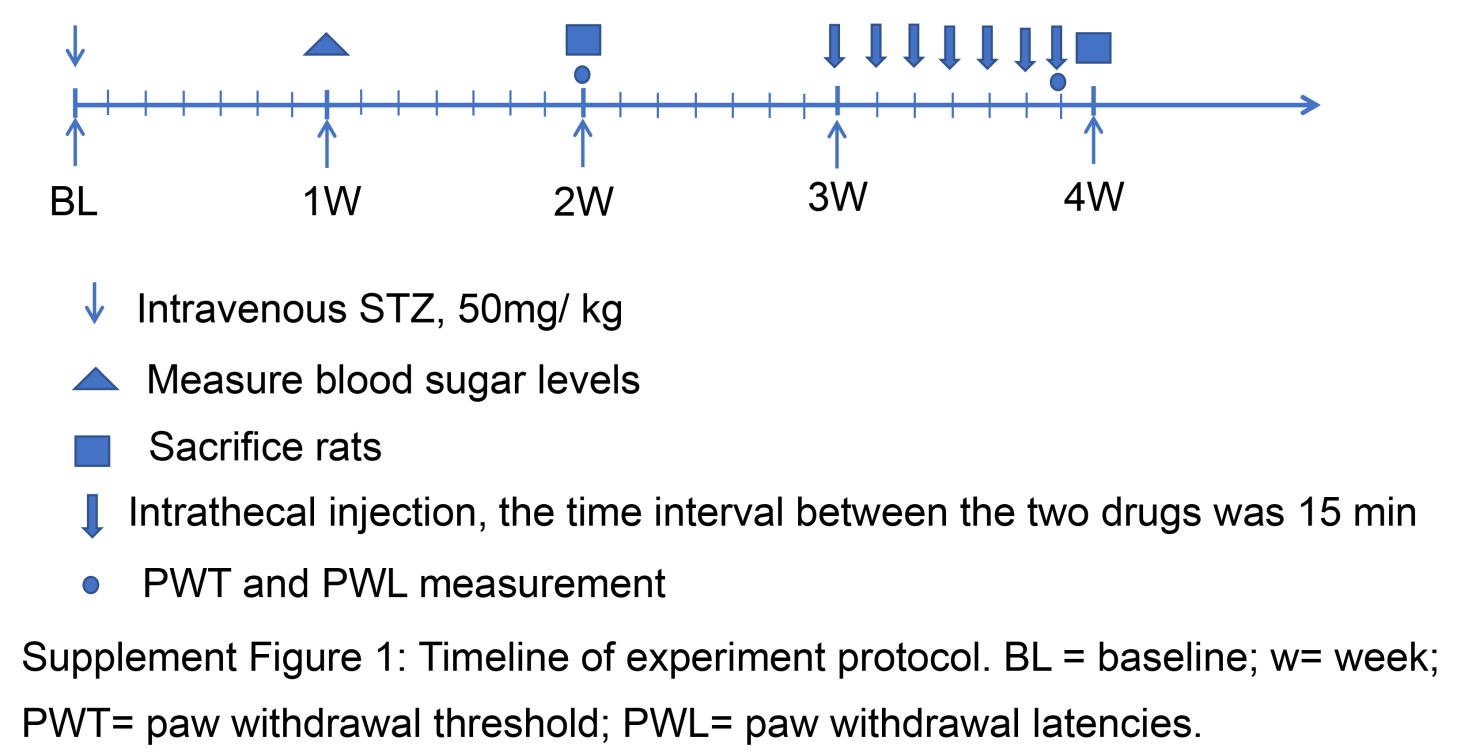
**

**Figure S1** The schematic diagram of the experimental process.

**
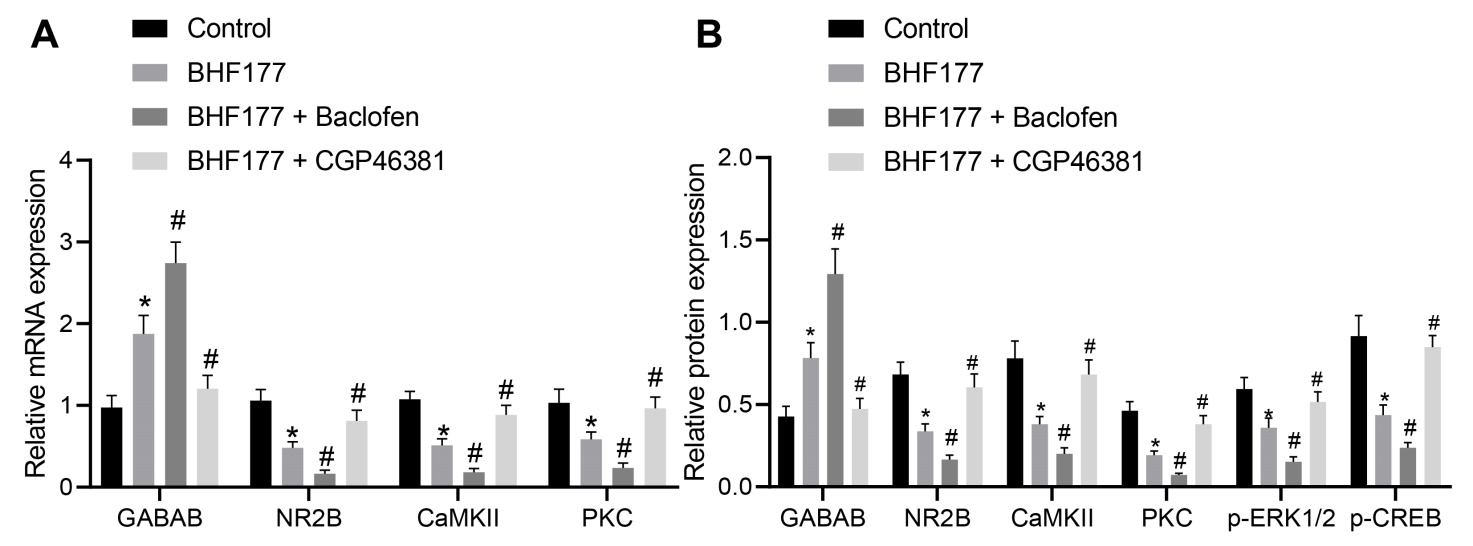
**

**Figure S2** BHF177 affects NR2B-PKC-CaMKII-ERK-CREB pathway in rat cerebellar nerve cells through GABAB. A, mRNA expression of GABAB, NR2B, PKC and CaMKII in neuronal cells determined by RT-qPCR. B, The protein expression of GABAB, NR2B, PKC, CaMKII, p-ERK1/2 or p-CREB in neuronal cells determined using immunoblotting. * *p* < 0.05 *vs.* normal neuronal cells, # *p* < 0.05 *vs.* BHF177 treatment. Cell experiment was repeated for three times.

**Supplementary Table 1 qRT-PCR primer sequence**

| Gene | Sequence |
| --- | --- |
| GABAB | Forward: 5′- AGCTCCAGTGATTTTAGGTCC -3′;  Reverse: 5′- TGACCACACAGCTTCCTAAC -3′ |
| PKC | Forward: 5′- TCTTCTACTGCTCTGACCCC -3′  Reverse: 5′- TCTGAGATCGGAAGGACAGG -3′ |
| CaMKII | Forward: 5′-TCACAGAGCCATCCCCGA -3′  Reverse: 5′-TGCTCTCAGAAGATTCCTTCAC -3′ |
| GAPDH | Forward: 5′- CTAGAGACAGCCGCATCTTC -3′  Reverse: 5′- CGTTGATGGCAACAATGTCC -3′ |

**Supplementary Table 2** The number of positive cells in GABAB receptor

| Groups | GABAB receptor positive cells |
| --- | --- |
| Normal rats | 35 ± 3.09 |
| Rat models of DNP | 15 ± 2.11 |
| Rat models of DNP + BHF177 | 30 ± 3.20 |
| Rat models of DNP + BHF177+ CGP4638 | 18 ± 2.49 |
